# The Frequency of Refractory Status Epilepticus and Its Outcome in a Tertiary Care Hospital in Pakistan: A Retrospective Study

**DOI:** 10.7759/cureus.29149

**Published:** 2022-09-14

**Authors:** Aisha Mansoor, Sahlish Kumar, Laraib Malik, Sufyan Razak, Reem Sulaiman, Qandeel Fatima, Faiza Zakaria, Ayman Iqbal, Farah Yasmin, Farheen Malik

**Affiliations:** 1 Pediatrics, Abbasi Shaheed Hospital, Karachi, PAK; 2 Internal Medicine, Dow University of Health Sciences, Karachi, PAK; 3 Internal Medicine, Sharif Medical and Dental College, Lahore, PAK; 4 Internal Medicine, Jinnah Sindh Medical University, Karachi, PAK; 5 Neurology, Dow University of Health Sciences, Karachi, PAK

**Keywords:** infections, developing country, pakistan, retrospective study, epilepsy

## Abstract

Background

Refractory status epilepticus (RSE) is a common neurologic emergency with refractory cases leading to increased rates of morbidity and mortality in patients. The lack of previous studies on the incidence, causes, and management of refractory status epilepticus in the pediatric population from our region prompted us to investigate further in this study.

Methods

We included retrospective data of all patients admitted to the pediatric intensive care unit (PICU) with a provisional diagnosis of RSE at a tertiary care hospital in Karachi from February 2019 to February 2021. No personal identification data was used, and confidentiality of the data was maintained throughout the analysis. The Statistical Package for the Social Sciences (SPSS) software version 22.0 (IBM SPSS Statistics, Armonk, NY, USA) was used to pool data and perform a descriptive analysis.

Results

Among the 687 patients who presented to the PICU with seizures, 50 (7.27%) patients were eventually diagnosed with RSE during the two-year period. The majority of the patients were male and less than one year of age. Infectious causes predominated our data cohort, and a four-drug regimen consisting of phenytoin, levetiracetam, valproic acid, and midazolam was able to terminate RSE in the majority of the patients in our setting (70%). The mortality rate was noted to be 22% among patients with RSE.

Conclusion

Morbidity and mortality among pediatric RSE patients are high in our settings. Urgent emergency services and timely cause-directed intervention could improve outcomes.

## Introduction

Status epilepticus (SE) is one of the most common life-threatening pediatric neurologic emergencies associated with significant morbidity and mortality. Early recognition, treatment, and determination of its etiology are vital parts of its management. Its definition has been modified over the years, but its conventional temporal evolution is divided into early/impeding (5-30 minutes), established (30-60 minutes), and refractory (≥60 minutes) phases [1,2]. SE can also be clinically divided into convulsive (CSE) and nonconvulsive (NCSE) types. CSE is defined as any continuous convulsive seizure activity or intermittent convulsive seizure activity without regaining consciousness between them lasting for more than five minutes [3]. The annual incidence of SE ranges from 10 to 73 per 100,000 people, with the highest incidence in children less than two years of age [4].

Refractory SE (RSE) is defined as unresolved clinical or electrographic seizure activity that is resistant to therapy with adequate doses of ≥2 anticonvulsant medications, one of which should be a benzodiazepine, or SE lasting 60 minutes or longer [2,5,6]. Approximately 10%-40% of patients with SE develop RSE with a mortality rate ranging from 16% to 43.5% [7-10]. The risk factors for SE to progress to RSE include, but are not limited to, acute symptomatic etiology (infections, electrolyte abnormalities, hypoxia, infarction, etc.), focal seizure at the onset of the episode, low Glasgow Coma Scale (GCS) score, NCSE, and higher peak serum glucose levels [8,11,12].

While the initial management of all cases of RSE remains the same, the etiology of RSE has been consistently linked to its outcome, and as such, it can vary with the region studied. There is a dearth of epidemiological studies on RSE in the pediatric population in our region. Therefore, this study aimed to retrospectively ascertain the etiology, frequency, associated risk factors, and outcome of RSE in pediatric patients in a tertiary care hospital in Karachi, Pakistan. This study will assist in better understanding the current demographics of patients presenting with RSE in our region and help guide further workup and follow-up of patients.

## Materials and methods

Study design and duration

A retrospective cross-sectional study was conducted at the pediatric intensive care unit (PICU) at Abbasi Shaheed Hospital, Karachi, Pakistan, a resource-limited tertiary care hospital. Two-year study data were obtained from February 2019 to February 2021.

Study population, inclusion criteria, and exclusion criteria

Data of every child admitted to the PICU with the provisional diagnosis of RSE was included in our study and obtained from medical records. No identifiable information was obtained from the records. The inclusion criteria consisted of the following: age between one month to 12 years, convulsive seizure at onset, and diagnosis of RSE, i.e., failure of two or more antiepileptics to terminate seizures or continuous infusion of any antiepileptic drug for seizure termination.

Exclusion criteria consisted of neonates, children already on antiepileptics, and children with an established or probable diagnosis of cerebral palsy. Records of such patients were excluded from the analysis. If any patient records were incomplete, they were also not included. Oral consent was taken from patients’ guardians for the use of data from clinical records during admission. Anonymity was maintained, and written consent was not required due to the retrospective nature of the study.

Drugs used for seizure control

As per the hospital protocol based on guidelines, children who presented with convulsive seizure activity for >5 minutes received three repeated doses of 0.3 mg/kg intravenous (IV) diazepam at 15-minute intervals along with a dose of 20 mg/kg of IV phenytoin (administered at a rate of 1 mg/kg/minute) [13].

If the seizures resumed or continued, they received IV levetiracetam 20 mg/kg for 10-30 minutes. If status epilepticus (SE) continued in children <2 years, 100 mg of pyridoxine was administered. If the seizure was not controlled after IV levetiracetam, IV valproic acid was added at a dose of 20-50 mg/kg.

If SE continued despite the first- and second-line medications (discussed above) or seizures lasted ≥60 minutes, the condition was considered as RSE, and the patient received IV midazolam at a loading dose of 0.3 mg/kg, followed by a continuous infusion of 1-5 μg/kg/minute titrated every 15 minutes. Treatment is typically for 24 hours. If control was still not achieved, thiopental sodium with an initial IV loading dose of 5 mg/kg and maintenance dose of 1-5 mg/kg/hour in the PICU was administered.

Patient records mentioned how long the seizure continued and which medicines were used for seizure termination.

Drug combination therapy definition in our study

Drug combination therapies employed at the hospital for the control and management of RSE are summarized as follows: three-drug therapy, phenytoin + levetiracetam + valproic acid; four-drug therapy, phenytoin + levetiracetam + valproic acid + midazolam; and five-drug therapy, phenytoin + levetiracetam + valproic acid + midazolam + thiopental sodium.

Study tool and statistical analysis

A precise performa was made to fill in details for the study from medical records. The performa consisted of baseline variables, including age group (<1 year or >1 year), gender (male or female), and vaccination status (complete, partial, or none). Other variables were related to the diagnosis of RSE, drug intervention, probable cause of RSE, and outcomes after RSE (survived, expired, or developed sequelae). The length of hospital stay (in days) was also noted at the end. No identifiable patient characteristics were used in this research.

The Statistical Package for the Social Sciences (SPSS) software version 22.0 (IBM SPSS Statistics, Armonk, NY, USA) was used to pool all the data and analyze it. Frequencies were calculated through descriptive statistics for demographic variables and outcome variables. Mean with standard deviation was calculated for the length of hospital stay.

## Results

Frequency of RSE

**Figure 1:**
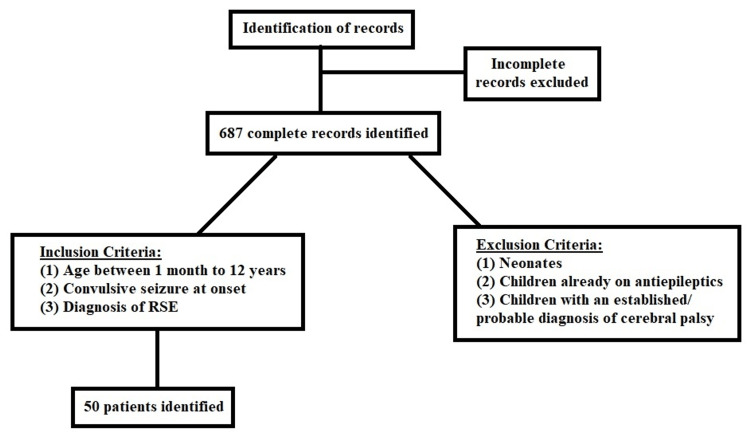
Inclusion of patients in the study. RSE: refractory status epilepticus

**Figure 2:**
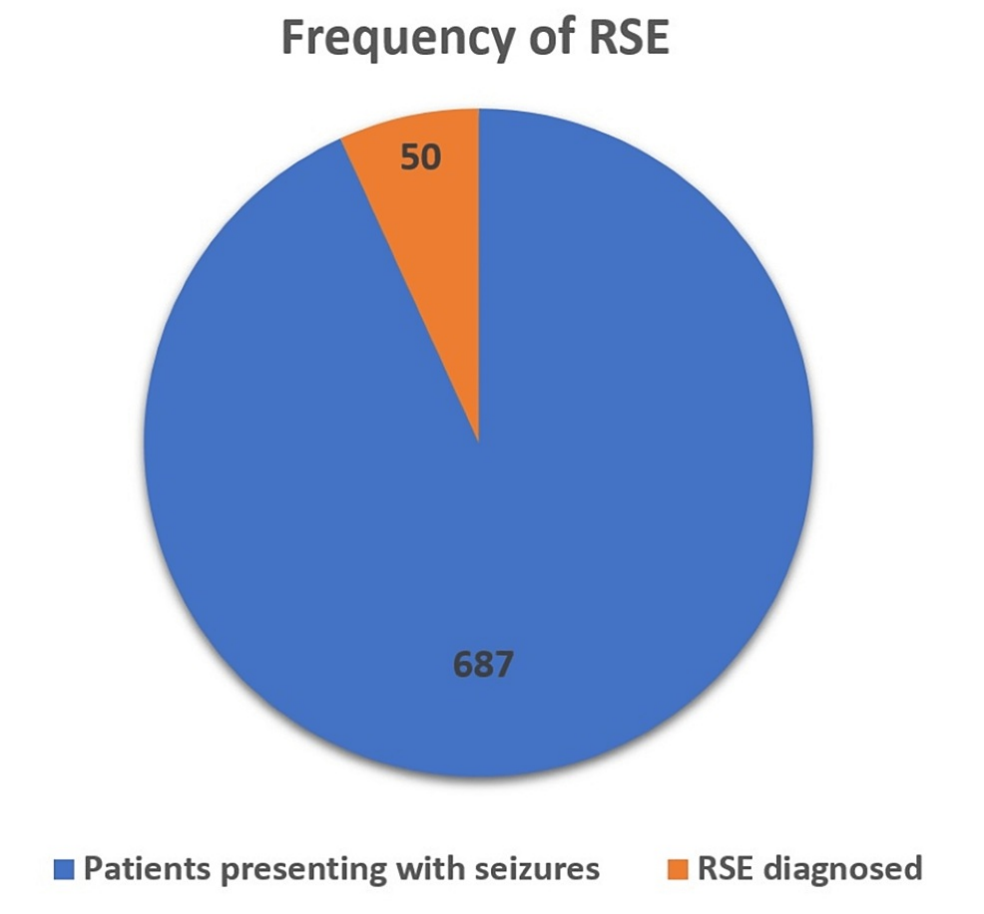
Frequency of RSE in patients who presented at the hospital in two years. RSE: refractory status epilepticus

A total of 687 patients were admitted to the PICU with seizures during the study period of two years. Based on exclusion and inclusion criteria, RSE was diagnosed in 50 patients (Figure 1). The frequency of RSE was found to be 7.27% in our study (Figure 2).

Baseline characteristics

The majority of the patients with RSE were males (n = 29, 58%). Patients under one year of age comprised 60% (n = 30) of the study population. Only nine (18%) RSE patients were completely vaccinated. The mean hospital stay of admitted RSE patients was 9 ± 2 days. Detailed baselines are presented in Table 1. The duration of seizure was 30 minutes in 12 (24%) patients, 30-60 minutes in 20 (40%) patients, and >60 minutes in 18 (36%) patients.

**Table 1:** Baseline characteristics of patients with RSE. RSE: refractory status epilepticus

Characteristics	Number of patients	Frequency (%)		p-value
Patients with RSE	50/687	7.27		-
Gender				
Males	29	58		<0.05
Females	21	42		
Age groups				
Age (<1 year)	30	60		<0.05
Age (≥1 year)	20	40		
Vaccination status				
Partial	18	36		<0.05
None/unvaccinated	23	46		
Complete	9	18		
Mean hospital stay	9 ± 2 days			-
Seizure duration				
30 minutes	12		24	-
30-60 minutes	20		40	-
>60 minutes	18		36	-

Causes of RSE

The most common probable cause of RSE in our patients was noted to be infections in 52% (n = 26), followed by magnetic resonance imaging (MRI) abnormality in 22% (n = 11) of the patients. Other probable causes mentioned in the patient notes included traumatic brain injury (history of trauma given at presentation), electroencephalography (EEG) abnormalities, suspected inborn errors of metabolism, and suspected neurodegenerative diseases. Infection was suspected based on physical examination findings and confirmed via lumbar puncture. Inborn errors of metabolism were suspected based on physical examination findings and family history.

EEG abnormalities were indicative of West syndrome in two patients (high amplitude, arrhythmic, and asynchronous electrical activity), subacute sclerosing panencephalitis (SSPE) in one patient (high amplitude delta waves that are repeated every four to seven seconds giving burst suppression), and acute disseminated encephalomyelitis (ADEM) in one patient (focal or generalized slowing and disturbance of normal sleep rhythms).

MRI abnormalities included meningeal enhancement (n = 3), microcephaly with ventriculomegaly (n = 3), white matter loss (n = 2), periventricular cysts (n = 2), and diffuse and poorly demarcated large (>1-2 cm) bilateral hyperintense lesions (n = 1).

**Figure 3:**
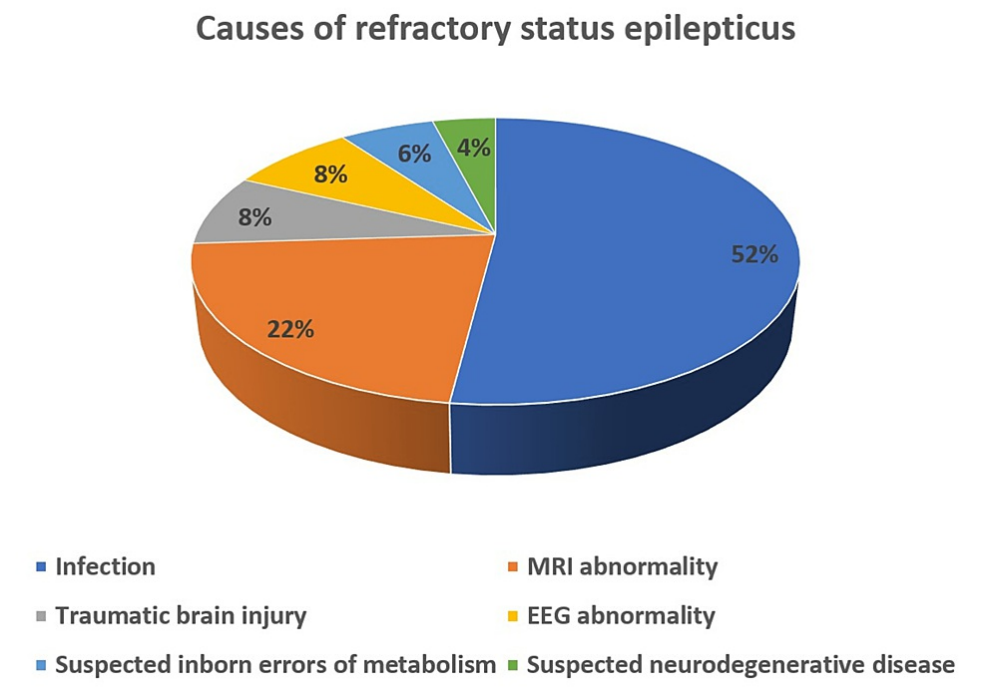
Causes of refractory status epilepticus in our study. MRI: magnetic resonance imaging; EEG: electroencephalogram

The details are mentioned in Table 2 and Figure 3.

**Table 2:** Probable causes of RSE in the patients. RSE: refractory status epilepticus; MRI: magnetic resonance imaging; EEG: electroencephalogram

Cause	Number of patients	Frequency (%)	Expired (n (%))
Suspected infection	26	52	7 (26.9)
MRI abnormality	11	22	2 (18.2)
Traumatic brain injury	4	8	-
EEG abnormality	4	8	-
Suspected inborn errors of metabolism	3	6	1 (33.3)
Suspected neurodegenerative disease	2	4	1 (50)

Drugs used to control RSE

After initial first-line and second-line drug regimens (mentioned in the methods section) failed to stop the seizures, the patient was termed RSE. Successful management of RSE was done using three-drug combination therapy in 24% of patients, four-drug combination therapy in 70%, and five-drug combination therapy in 6%. Table 3 shows the details of drug therapy.

**Table 3:** Drugs used for controlling refractory status epilepticus.

Drug therapy	Drugs used	Number of patients	Frequency (%)
Three-drug therapy	Phenytoin + levetiracetam + valproic acid	12	24
Four-drug therapy	Phenytoin + levetiracetam + valproic acid + midazolam	35	70
Five-drug therapy	Phenytoin + levetiracetam + valproic acid + midazolam + thiopental sodium	3	6

Outcome of RSE

Out of 50 patients, most of the patients survived and were discharged (n = 34, 68%). The details of patients who expired and developed sequelae are mentioned in Table 4. The majority of the patients (7/11) who expired had infective causes; out of them, three patients refused lumber puncture and denied further workup (mentioned in records). There was no significant association of the outcomes of RSE with age, gender, vaccination status, and probable cause of RSE (p > 0.05).

**Table 4:** Outcome of patients with refractory status epilepticus.

Outcome	Number of patients	Frequency (%)
Survived	34	68
Expired	11	22
Developed sequelae (e.g., cerebral palsy, stroke, and cranial nerve palsies)	5	10

## Discussion

Our study demonstrates the incidence, clinical characteristics, management, and outcomes of children who presented with RSE. Our results reveal a low overall incidence rate, infection as the leading cause, and high mortality of patients with RSE.

Incidence and characteristics

The results show that around 7.27% of patients who presented with convulsions were diagnosed with RSE, which lies below the average range of 10%-40% reported by previous studies but much higher than Schubert-Bast et al. have documented (1.07%) [7,8,14]. The different incidence rates reported in studies can be attributed to the sociological, economic, and geographical diversity of the study populations and the referral bias existing in their selection, and the lack of a standard definition for RSE. In our study, 58% of patients were male, and the majority were less than one year of age. Only 18% of patients were completely vaccinated; however, there was no significant association between outcome and age, gender, or vaccination status (p > 0.05). The mean hospital stay of patients in our cohort was 9 ± 2 days, which was less than the average 20.2 days reported in Germany; however, we have not stratified the length of hospital stay with the etiology as that significantly impacts the management and in-hospital care needed for patients [14,15]. Since our hospital did not have a dedicated pediatric neurology department, many patients were referred to other hospitals for better management, and hence, a shorter hospital stay is noted in our study.

Etiology

The cause of RSE has been repeatedly shown to affect morbidity, duration of seizure, and response to treatment [15-17]. Infections resulted in a significant proportion of RSE cases in our study (52%), followed by abnormalities detected on MRI (22%). Previous studies from different geographical regions and socioeconomic backgrounds have mainly reported cryptogenic (>50%) and autoimmune as the leading causes of RSE [18,19]. The difference in etiologies has been shown to be age-dependent and vary even among children of different ages [20,21]. Our study data are from a public sector tertiary care hospital. Therefore, the patients’ low overall socioeconomic status and unsanitary living conditions can impact the infection rates and RSE rates.

Management

Therapy for RSE is aimed at seizure control, neuroprotection, and sequela prevention. After the failure of benzodiazepines and antiepileptics to control the seizure activity, coma-inducing agents (“aggressive therapy”) are commonly used to achieve electrographic normalization or burst suppression, which should be maintained for at least 1-2 days before weaning off of therapy. The various agents used and their level of evidence are summarized by Vasquez et al. [22]. In our study, all patients achieved control with three to five drug regimens, most of which responded to the four-drug regimen (Table 3). Infusion of midazolam (part of the four-drug regimen), a fast-acting benzodiazepine, achieved cessation of seizure activity in 70% of patients. However, since there is no standardized drug regimen for RSE treatment, which is individualized according to patient response, neurologist’s/pediatrician’s discernment, and hospital protocols, it is not possible to compare the results from our study with previous reports.

Outcome

The outcome of children with RSE is poor in our setting, with 22% mortality. Previous studies have shown a wide range of mortality [9,10]; however, Raspall-Chaure et al. have shown that mortality is associated with the causative factor and not necessarily the seizure itself. Meningoencephalitis was associated with particularly high mortality, and this can explain the high mortality rate in our study. Although the age at onset of RSE affects outcome, we did not find any significant association between the two [17].

Previous studies have shown that delay in treatment and longer duration of RSE contributed to worse clinical outcomes [23-25]. Therefore, prompt diagnosis and initiation of anticonvulsive therapy along with robust investigation to ascertain the etiological cause with its subsequent early management can reduce morbidity and mortality rates.

Our study has limitations. Our study is retrospective in nature and based on data from a single center; more widespread studies are needed. Due to the small number of patients, associations among different variables could not be established. We were unable to determine if the causative agent of infectious etiology was preventable with vaccination. The study also did not assess for any non-pharmacological therapies used. Due to the retrospective nature of the study, we could not establish if patients had any recurrent episodes of RSE. Due to the small sample size, the type of epilepsy and its correlation with EEG and MRI findings, and its further relation with the treatment or drug therapy as well as the outcome could not be established. A prospective, cross-sectional study spanning over multiple centers is needed to provide a more generalized estimate.

## Conclusions

Infectious diseases predominate as the cause of RSE in children in our region. The majority of RSE patients in our study were managed on four-drug therapy (phenytoin + levetiracetam + valproic acid + midazolam). The mortality rate in children diagnosed with RSE remains high in our patients, and many patients develop sequelae such as cranial nerve deficits. Urgent determination of causative etiology and rapid initiation of therapy can lower morbidity and mortality rates. Proper and timely administration of vaccines could potentially curb infections leading to RSE and subsequent morbidity. Metabolic disorders require prompt diagnosis, but due to the cost related to the tests and limited availability, diagnosis is delayed or sometimes never made, and the patient expires. Lack of awareness further exacerbates the conditions as patients present after a substantial amount of time has passed and seizures have not stopped; this can worsens the prognosis. Parents sometimes ignore a single seizure episode, and a diagnosis of epilepsy is made in some children when they present with status epilepticus. More multicenter, longitudinal studies are needed to ascertain the magnitude of RSE burden and associated risk factors. Further studies are required to quantify the actual impact of interventions such as adequate vaccination in preventing RSE.

## References

[1] Samanta D, Garrity L, Arya R (2020). Refractory and super-refractory status epilepticus. Indian Pediatr.

[2] Sánchez Fernández I, Abend NS, Agadi S (2014). Gaps and opportunities in refractory status epilepticus research in children: a multi-center approach by the Pediatric Status Epilepticus Research Group (pSERG). Seizure.

[3] Lowenstein DH, Bleck T, Macdonald RL (1999). It's time to revise the definition of status epilepticus. Epilepsia.

[4] Singh RK, Gaillard WD (2009). Status epilepticus in children. Curr Neurol Neurosci Rep.

[5] Falco-Walter JJ, Bleck T (2016). Treatment of established status epilepticus. J Clin Med.

[6] Brophy GM, Bell R, Claassen J (2012). Guidelines for the evaluation and management of status epilepticus. Neurocrit Care.

[7] Lewena S, Young S (2006). When benzodiazepines fail: how effective is second line therapy for status epilepticus in children?. Emerg Med Australas.

[8] Barzegar M, Mahdavi M, Galegolab Behbehani A, Tabrizi A (2015). Refractory convulsive status epilepticus in children: etiology, associated risk factors and outcome. Iran J Child Neurol.

[9] Sahin M, Menache CC, Holmes GL, Riviello JJ (2001). Outcome of severe refractory status epilepticus in children. Epilepsia.

[10] Kim SJ, Lee DY, Kim JS (2001). Neurologic outcomes of pediatric epileptic patients with pentobarbital coma. Pediatr Neurol.

[11] Mayer SA, Claassen J, Lokin J, Mendelsohn F, Dennis LJ, Fitzsimmons BF (2002). Refractory status epilepticus: frequency, risk factors, and impact on outcome. Arch Neurol.

[12] Tatro HA, Hamilton LA, Peters C, Rowe AS (2020). Identification of risk factors for refractory status epilepticus. Ann Pharmacother.

[13] Glauser T, Shinnar S, Gloss D (2016). Evidence-based guideline: treatment of convulsive status epilepticus in children and adults: report of the guideline committee of the American Epilepsy Society. Epilepsy Curr.

[14] Schubert-Bast S, Zöllner JP, Ansorge S (2019). Burden and epidemiology of status epilepticus in infants, children, and adolescents: a population-based study on German health insurance data. Epilepsia.

[15] Metsäranta P, Koivikko M, Peltola J, Eriksson K (2004). Outcome after prolonged convulsive seizures in 186 children: low morbidity, no mortality. Dev Med Child Neurol.

[16] Lowenstein DH (1999). Status epilepticus: an overview of the clinical problem. Epilepsia.

[17] Raspall-Chaure M, Chin RF, Neville BG, Scott RC (2006). Outcome of paediatric convulsive status epilepticus: a systematic review. Lancet Neurol.

[18] Gaspard N, Foreman BP, Alvarez V (2015). New-onset refractory status epilepticus: etiology, clinical features, and outcome. Neurology.

[19] Husari KS, Labiner K, Huang R, Said RR (2020). New-onset refractory status epilepticus in children: etiologies, treatments, and outcomes. Pediatr Crit Care Med.

[20] Shinnar S, Pellock JM, Moshé SL (1997). In whom does status epilepticus occur: age-related differences in children. Epilepsia.

[21] Jafarpour S, Hodgeman RM, De Marchi Capeletto C, de Lima MT, Kapur K, Tasker RC, Loddenkemper T (2018). New-onset status epilepticus in pediatric patients: causes, characteristics, and outcomes. Pediatr Neurol.

[22] Vasquez A, Farias-Moeller R, Tatum W (2019). Pediatric refractory and super-refractory status epilepticus. Seizure.

[23] Gaínza-Lein M, Sánchez Fernández I, Jackson M (2018). Association of time to treatment with short-term outcomes for pediatric patients with refractory convulsive status epilepticus. JAMA Neurol.

[24] Sánchez Fernández I, Abend NS, Agadi S (2015). Time from convulsive status epilepticus onset to anticonvulsant administration in children. Neurology.

[25] Claassen J, Lokin JK, Fitzsimmons BF, Mendelsohn FA, Mayer SA (2002). Predictors of functional disability and mortality after status epilepticus. Neurology.

